# Association Between Shopping Assistance and Functional Decline in Older Residents with Support Levels Under the Long-Term Care Insurance System in Japan: A Retrospective, Cross-Sectional Study

**DOI:** 10.3390/geriatrics9060162

**Published:** 2024-12-14

**Authors:** Akihiko Asao, Toshimasa Sone, Takaaki Fujita, Hiroshi Hayashi, Shigeki Kurasawa, Koshi Sumigawa, Yohko Ishikawa, Hironori Kawamata, Yuhei Mitsuhashi, Yoshinobu Tanaka, Natsumi Kimura, Kazuaki Iokawa

**Affiliations:** Department of Occupational Therapy, School of Health Sciences, Fukushima Medical University, Fukushima 960-8516, Japan

**Keywords:** community-dwelling older adults, long-term care insurance, shopping assistance, functional decline, disability

## Abstract

**Background/Objectives:** Maintaining functional independence and minimizing disability among older adults living in the community is paramount for mitigating rising care demands. Our study focused on shopping as a critical instrumental activity of daily living (ADL) to explore the association between shopping assistance and functional decline among older individuals receiving support through long-term care insurance (LTCI). **Methods:** This retrospective, cross-sectional study included 6202 participants aged >65 years living in a Japanese regional town receiving LTCI support, suggesting that they required assistance with local community life. Logistic regression analysis identified several factors associated with shopping assistance among the participants, including physical and cognitive functions, functional ADL, and psychobehavioral symptoms. **Results:** In male participants, walking dysfunction, short-term memory decline, decreased frequency of going outdoors, and decreased engagement in personal grooming were significantly associated with requiring shopping assistance. Conversely, in female participants, reduced physical function and walking performance were significantly associated with requiring shopping assistance, whereas dependence on personal grooming was less pronounced than in male participants. **Conclusions:** These findings suggest that, in addition to direct shopping assistance, tailored interventions targeting physical, cognitive, and ADL functions—while considering gender-specific needs—may help older adults maintain independence in shopping activities as part of their daily community life.

## 1. Introduction

In Japan, the growing aging population is a major societal concern [1], and disability prevention programs are becoming increasingly important [2]. These programs have two primary goals: preventing functional decline and halting the progression of disability among community-dwelling older adults [2]. The Japanese government actively promotes measures to reduce the need for care support. The Long-Term Care Insurance (LTCI) system, introduced in 2000, aims to support older individuals with disabilities in their social lives. The LTCI classifies individuals into seven care levels: support levels 1–2 and care levels 1–5, ranging from mild to severe disabilities. Although those certified at the care level require support for basic activities of daily living (ADLs), older individuals certified at the support levels can perform basic ADLs and need assistance with instrumental ADLs (IADLs) [3]. To prevent the further progression of disability in older adults under LTCI support levels, identifying the ADL or IADL tasks for which they are most likely to need care is crucial.

Shopping could be a key indicator for preventing further disabilities in community-dwelling older adults. According to a report from the Japanese Ministry of Health, Labor, and Welfare, shopping was identified as a daily activity for which older adults would most likely require support [4]. Furthermore, recent European studies have revealed that shopping disability occurs before dependency in basic ADL [5] and is negatively correlated with quality of life and survival in older individuals [6,7]. Consequently, focusing on older adults certified as requiring support level 1 or 2 in the LTCI system—who are generally independent in basic ADLs but require partial support for IADLs—may provide valuable insights into the early stages of functional decline and related health issues. These insights are crucial not only for planning effective shopping assistance but also for developing comprehensive health support strategies for this population.

However, the evidence on the functional and disability status of community-dwelling older adults who require assistance with shopping is limited. Previous studies have identified various factors related to shopping assistance, including poor health [8,9,10], dietary habits [11,12], personal factors [8,9,11,12], environmental barriers [10,11,12], and functional decline [8,9,10,12,13]. These studies suggest that a wide range of functional and environmental factors may influence the need for shopping assistance in older adults, though the evidence remains limited. Notably, aspects such as cognitive function, psychobehavioral status, and broader functioning, including ADLs and disabilities, have not been thoroughly examined in this context. Therefore, the full scope of functional and disability status—from body functions and structures to activities and participation—has not been clearly identified in older adults who require shopping assistance, leaving unanswered questions about how functional declines and disabilities manifest in their daily lives.

Additionally, more evidence is needed on sex differences in shopping-related care among community-dwelling older adults. Sex differences are a common theme in gerontology, encompassing life expectancy, mortality, morbidity, health-related behaviors, and subjective well-being [14,15,16]. Although some studies have examined the factors associated with shopping assistance in both men and women, sex differences in functional and disability status in daily life have rarely been explored [11,12]. Moreover, the specific nature of these sex differences remains unclear [8,9,10,11,12,13].

In the present study, we examined the association between shopping assistance, functioning status, and disability among community-dwelling older men and women certified at support levels in the LTCI system. The objective was to explore potential relationships between the need for shopping assistance and other functional declines in this population. We hypothesized that, in addition to physical functions, cognitive, psychobehavioral, and ADL functions would be associated with the need for shopping assistance among older adults. Furthermore, we hypothesized that older adults requiring shopping assistance might exhibit different patterns of functional declines, depending on their sex. This retrospective, cross-sectional study utilized LTCI certification data from Koriyama City, a regional area in Japan. The LTCI certification checklist includes a wide range of components related to functioning and disability. Older adults classified under LTCI support levels may require some assistance with IADLs, particularly shopping, while remaining capable of self-care. Therefore, we analyzed the certification data of older adults classified under LTCI support levels.

## 2. Materials and Methods

### 2.1. Study Design and Participants

**Figure 1:**
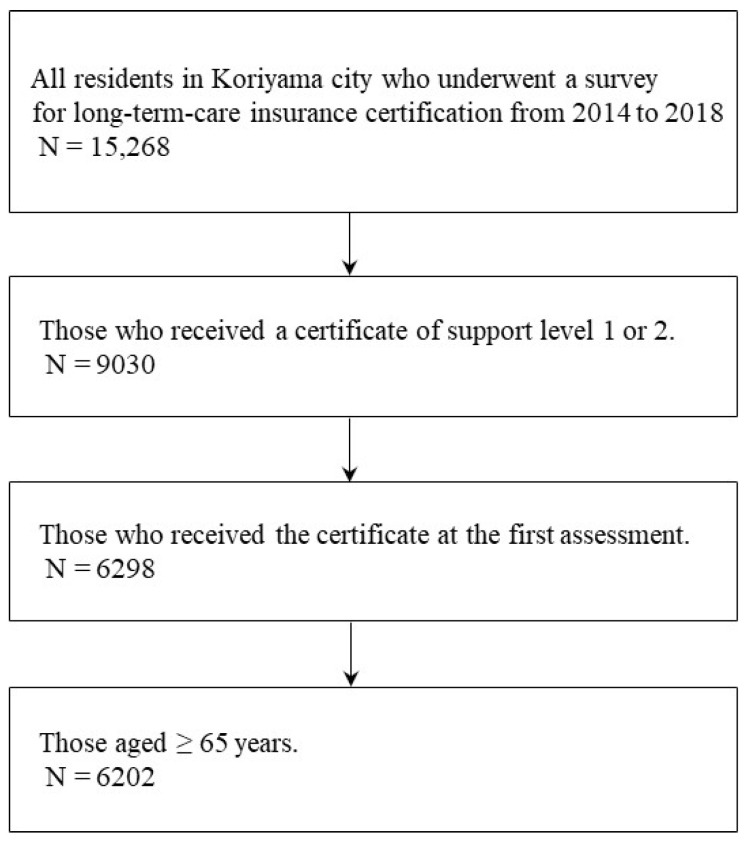
Flowchart of the study participants.

This retrospective, cross-sectional study was conducted as part of a collaborative research project between Koriyama City, Fukushima Prefecture, Japan, and Fukushima Medical University. This study contributes to the Sustainable Development Goals Experiential Future City Koriyama Cross-Generational Project by focusing on the development of healthier cities. Koriyama City, with over 300,000 residents, includes both urban and rural areas. In 2016, the midpoint of the data collection period, the aging rates of individuals over 65 years in Koriyama City and Japan were 29.0% and 27.3%, respectively [17,18]. We analyzed the data of 15,268 people in Koriyama City who were certified as requiring long-term care between 2014 and 2018. From this pool, we recruited 6202 residents who had been assessed for long-term care needs, were certified as requiring level 1 or 2 support, had received their first certification during this period, and were aged over 65 years (Figure 1).

### 2.2. Measurements

We used the assessment data from LTCI certification in Koriyama City, collecting information such as that pertaining to sex and age from the profile section and 50 components from the certification checklist related to mental and physical health conditions from the basic assessment section. These components comprised (1) 13 items related to physical function, (2) 12 items related to ADL function, (3) nine items related to cognitive function, (4) 15 items related to psychiatric and behavioral symptoms, and (5) one item related to adaptation to social life. Shopping was the only item included in Section 5. The other 47 items related to functional decline and disability were covered in Section 1, Section 2, Section 3 and Section 4.

During the assessment process, a government worker or certified inspector in healthcare services interviewed older adults who had applied for long-term care to ascertain their symptom presentation and level of dependence. For instance, regarding shopping assistance, the questionnaire instructed the evaluator to choose one of the following four options that best described their need for shopping assistance: independence (ability to shop unassisted), supervision (requiring caregiver oversight while shopping), partial assistance (needing help from a caregiver while shopping), or total assistance or inability to go shopping independently. For analysis, the four degrees of assistance were grouped under the categories of “independent” (no assistance) and “dependent” (supervision, partial assistance, and total assistance).

### 2.3. Statistical Analysis

Before starting data analysis, we converted the data into dichotomous variables. For each item, the response indicating the best condition—signifying independence, ability, or absence of symptoms—was coded as “Zero”, whereas all other responses were coded as “One”, thereby transforming ordinal scales into dummy variables [19]. Age data were categorized into three levels: 65–74, 75–84, and >85 years, with 65–74 years as the reference category. Sex data were coded as “Zero” for the male sex and “One” for the female sex, with the male sex as the reference category. First, chi-square tests or Fisher’s exact tests, as appropriate, were applied to examine differences in item responses between individuals who are independent in shopping and those who require assistance. Next, we explored the potential relationship between shopping assistance and functional decline and disabilities. To this end, multiple logistic regression analysis using the stepwise method was performed to calculate odds ratios (ORs) with 95% confidence intervals (CIs) for factors associated with functional decline and disabilities, as well as the need for shopping assistance (significance levels for entry = 0.05 and stay = 0.10). Shopping assistance served as the dependent variable, whereas functioning and disability levels (physical function, ADL function, cognitive function, and psychiatric and behavioral symptoms) and demographic factors (age and sex) were included as independent variables because of the exploratory nature of this study. Statistical analyses were conducted for all participants in the first step and for each sex in the second step. The model’s goodness of fit was evaluated using the Hosmer–Lemeshow test. In addition, a multinomial logistic regression analysis was conducted as a sensitivity analysis. The dependent variable was shopping assistance, which was categorized into three levels based on the data distribution: independent (N = 1631), supervision-partial assistance (N = 788), and total assistance (N = 3783). In this analysis, “independent” was used as the reference category. The explanatory variables included items from the LTCI certification survey, age, and sex, with the best condition (e.g., independent, no symptoms) serving as the reference category. All statistical analyses were performed using SAS software (version 9.4; SAS Institute, Cary, NC, USA), and *p*-values < 0.05 were considered statistically significant.

### 2.4. Ethical Considerations

The present study’s dataset was obtained from Koriyama City, Japan, and anonymized prior to our access. Owing to the anonymized nature of the data, the requirements for individual informed consent were waived. The dataset was accessed on 15 September 2023. This study was approved by the Fukushima Medical University Ethics Committee (General 2021-105).

## 3. Results

### 3.1. Participant Characteristics and Health Conditions

A total of 6202 residents of Koriyama City were included in the analysis, comprising 4300 participants (69.3%) at support level 1 and 1902 (30.7%) at support level 2. Additionally, 1631 participants were independent in shopping (26.3%), and 4571 participants were dependent in shopping (73.7%).

Among the participants with independent shopping status, 1251 were certified at support level 1 (76.7%), whereas, among the participants with dependent status, 3049 were certified at support level 1 (66.7%). The health conditions of both groups are detailed in Table 1.

### 3.2. Association Between Shopping Assistance and Functional Decline

In the initial analysis, variables such as age, sex, various physical functions, ADL functions, cognitive functions, and psychiatric and behavioral symptoms were associated with shopping assistance for all participants. Various health condition-related factors were associated with shopping assistance (Table 2). Notably, only sex was negatively related to shopping independence (OR = 0.51, 95% CI = 0.44–0.59), indicating that female participants were less likely to require shopping assistance compared to male participants. The Hosmer–Lemeshow test was non-significant (*p* = 0.06), indicating an adequate model fit.

The results of multiple logistic regression analyses for male and female participants are presented in Table 3 and Table 4, respectively. For male participants, factors such as older age (age 75–84 years: OR = 1.50, 95% CI = 1.07–2.10; age > 85 years: OR = 1.80, 95% CI = 1.29–2.49), physical functioning decline (walking without holding: OR = 1.72, 95% CI = 1.30–2.27; cutting nails: OR = 1.44, 95% CI = 1.04–1.99; acuity: OR = 1.95, 95% CI = 1.10–3.43), disability (oral hygiene: OR = 10.76, 95% CI = 1.45–80.00; washing face: OR = 4.36, 95% CI = 1.02–18.70; dressing lower body: OR = 9.74, 95% CI = 1.31–72.51; going outdoors: OR = 8.82, 95% CI = 6.19–12.58), cognitive decline (short-term memory: OR = 2.53, 95% CI = 1.49–4.29), and psychological symptoms (severe memory loss: OR = 1.32, 95% CI = 1.02–1.71) were associated with shopping assistance. The Hosmer–Lemeshow test was non-significant (*p* = 0.80), indicating an adequate model fit.

For female participants, older age (age > 85 years: OR: 1.49, 95% CI = 1.28–1.75), physical functioning decline (standing with feet without holding: OR = 1.43, 95% CI = 1.12–1.83; walking without holding: OR = 1.42, 95% CI = 1.16–1.74; standing on one leg without holding: OR = 1.55, 95% CI =1.28–1.89; washing body: OR = 1.86, 95% CI = 1.46–2.37; cutting nails: OR = 2.22, 95% CI = 1.79–2.75), disability (washing face: OR = 4.87, 1.51–15.68; going outdoors: OR = 6.95, 95% CI = 5.58–8.66), cognitive decline (expressing one’s intention: OR = 6.73, 95% CI = 1.94–23.39; short-term memory: OR = 3.38, 95% CI = 2.42–4.73), and psychological symptoms (repeating the same story: OR = 1.82, 95% CI = 1.30–2.54) were associated with shopping assistance. The Hosmer–Lemeshow test was significant (*p* = 0.01), suggesting a less ideal model fit.

The significant variables identified in the multinomial logistic regression analysis are provided in the Supplementary Materials. This additional analysis confirmed the robustness of the primary results. Notably, all items had variance inflation factor values below 2.0, indicating no multicollinearity among the explanatory variables.

## 4. Discussion

In this study, we explored the association between shopping assistance and factors related to functioning and disability among older residents classified under LTCI support levels in Koriyama City, Japan. Comprehensive data analysis revealed that a decline in physical, ADL, cognitive, and behavioral and psychological functions was associated with shopping assistance, suggesting that shopping involves many processes that require adequate physical and cognitive abilities, as well as ADL functions. Specifically, maintaining lower extremity function and walking performance is crucial for older residents to prevent needing shopping assistance, aligning with the findings of previous studies in which shopping difficulties were associated with the decline in lower extremity function and walking performance [8,9,11]. A recent meta-analysis showed that worsening IADLs were associated with reduced muscle mass, hand grip strength, and gait speed [20]. Moreover, decreased walking speed was a predictor of disability in IADLs, including shopping, among community-dwelling older adults in European countries [21]. Thus, maintaining lower extremity functions and walking performance is important for older adults so that they can shop independently at local stores.

Additionally, this study highlights the importance of cognitive function in independent shopping. Cognitive functioning is responsible for planning schedules, creating shopping lists, managing money, and other tasks. The present results across total, male, and female participants indicated short-term memory, expressing one’s intentions, recalling the current season in cognitive function, and repeating the same story, and severe memory loss in psychobehavioral symptoms was associated with the need for shopping assistance. Previous studies on older adults have identified declines in executive function and short-term memory as predictors of impairments in IADLs, including shopping [22,23,24,25,26]. Cognitive assessments, such as the Mini-Mental State Examination [27] and the Revised Hasegawa’s Dementia Scale [28], have identified components like short-term memory and seasonal orientation as predictors of dependence in shopping tasks [22,23], findings that closely align with ours. These cognitive abilities directly impact shopping tasks, for which memory supports item recall and seasonal awareness assists in planning relevant purchases. However, it is important to note that the LTCI system’s certification checklist does not assess executive function, which limited our study from evaluating the relationship between executive dysfunction and the need for shopping assistance.

Moreover, this study reports a novel finding, namely that shopping assistance is associated with various disabilities of daily life among local-dwelling older residents classified under LTCI support levels. ADL functions, such as bathing, cutting nails, eating, oral hygiene, urination, washing the face, and dressing the lower limbs, which are crucial personal grooming activities that are carried out before going outdoors, were linked to shopping assistance, suggesting that shopping assistance may be connected to a decline in personal grooming behavior among older residents classified under LTCI support levels. For community-dwelling older people, cutting nails, a basic ADL, is as challenging as shopping [3,29,30,31]. The results underscore the need for preventive care services that address not only independence in shopping but also the personal grooming of users with LTCI support status.

Our findings indicate a gender effect, with men showing a stronger association with shopping assistance needs compared to women. When analyzed by gender, we observed distinct variations in the functional decline factors associated with shopping assistance. Many gerontological studies have reported that women are generally more prone to ADL declines than men [32,33,34,35,36]. However, our analysis of the total participant pool revealed that being female was negatively correlated with the need for shopping assistance, suggesting that men in our study were more likely to require support. Although this finding appears to contrast with previous reports [32,33,34,35,36], similar results were observed in a study using LTCI system certification data, which found that men had greater assistance needs across several ADLs [31]. This aligns with our finding that men demonstrated a stronger association with shopping assistance overall and exhibited more ADL-related variables in gender-specific analyses. Additionally, a Canadian study on community-dwelling older adults found that men were more likely to need shopping assistance, possibly because of traditional household roles [9]. Although our study did not include data on household roles or social participation, these factors may partially explain why male participants in our study required shopping assistance and showed more ADL impairments. In the gender-specific analysis, we found that, among older male participants, the need for shopping assistance was associated with declines in walking ability, memory, the frequency of outdoor activities, and personal grooming behaviors, such as cutting nails, oral hygiene, washing the face, and dressing the lower body. In contrast, older female participants exhibited associations with walking dysfunction, declines in short-term memory, difficulties in expressing intentions, and a decreased frequency of outdoor activities. Additionally, females demonstrated physical function declines, including difficulty standing unsupported and standing on one leg (Table 3 and Table 4).

When planning preventive care for older adults who require only minimal support, it is essential to offer not only direct shopping assistance but also targeted interventions that address the functional declines associated with shopping assistance, as identified in this study. A preliminary study by Mouri et al. [37] reported the preventive care effect of shopping rehabilitation for community-dwelling older individuals, emphasizing the importance of the physical and cognitive components related to shopping in grocery stores. Our findings provide further insights, suggesting that, in preventive shopping rehabilitation programs, older male users should focus on their personal grooming and appearance in preparation for shopping, whereas female users should concentrate on improving their lower extremity function and walking performance.

This study involved some limitations. First, the generalizability of the findings is limited, as we used data from LTCI certification in a single regional city in Japan. Therefore, the findings may not fully represent other regions in Japan or countries with different long-term care systems. However, because the data are derived from the entire LTCI certification survey for the city, this study offers valuable insights with minimal selection bias. Second, our model construction and validation were constrained due to limited incorporation of comprehensive factors such as poor health [8,9,10], dietary habits [11,12], personal factors [8,9,11,12], and environmental barriers [10,11,12], all of which may influence the need for shopping assistance. The factors affecting shopping assistance among community-dwelling older adults are complex and multifaceted. The lower model fit observed in the data of female participants suggests that future research could improve model accuracy by integrating additional background factors, such as environmental and personal variables, and accounting for unknown error variables. To develop more robust and comprehensive models, future studies could employ advanced multivariate analysis techniques, such as structural equation modeling, to better capture the complexity of factors influencing the need for shopping assistance. Finally, future studies could benefit from a longitudinal approach and a larger dataset that includes a broader range of variables to enhance the robustness and applicability of the findings.

## 5. Conclusions

This study explored the association between shopping assistance and factors related to functioning and disability among older residents classified under LTCI support levels. The findings revealed that shopping assistance needs are associated not only with physical functions, such as standing and walking ability, but also with cognitive functions, including short-term memory, and ADL functions, such as cutting nails, washing the face, and going outdoors. Furthermore, our study identified sex-specific characteristics in the need for shopping assistance. Among males, shopping assistance needs were associated with walking function, short-term memory, reduced frequency of outdoor activities, and lower engagement in grooming behaviors. In contrast, female participants requiring shopping assistance exhibited more pronounced declines in physical functions but less decline in grooming behaviors compared to males. These findings emphasize the importance of providing not only direct shopping assistance but also targeted interventions for the functional declines associated with shopping needs. Specifically, considering gender-specific needs may support older adults in maintaining independence in shopping activities as part of their daily lives.

## Figures and Tables

**Table 1:** Participant characteristics and health conditions.

	Shopping		
	Independent N = 1631	Dependent N = 4571	*p*-Value
Age (years), %			
65–74	13.2	12.0	<0.01
75–84	44.7	35.9	
>85	42.1	52.1	
Sex, %			
Male	25.7	39.0	<0.01
Health condition and functional activities, %			
(1) Physical functions			
Paralysis, none (absence)	74.9	69.6	<0.01
Contracture, none (absence)	88.9	88.3	0.54
Rolling over in bed without holding, possible	75.3	63.6	<0.01
Sitting up in bed without holding, possible	1.4	1.3	0.62
Sitting without holding, possible	80.0	70.8	<0.01
Standing with feet without holding, possible	88.8	74.1	<0.01
Walking without holding, possible	71.7	51.5	<0.01
Sitting to standing without holding, possible	3.9	3.1	0.09
Standing on one leg without holding, possible	38.3	21.9	<0.01
Washing body, independent	90.2	72.6	<0.01
Cutting nails, independent	87.5	69.5	<0.01
Acuity, normal (nothing wrong)	96.9	93.8	<0.01
Hearing, normal (nothing wrong)	73.2	64.3	<0.01
(2) ADL functions			
Transfer, independent	100.0	99.7	0.03
Mobility, independent	99.8	99.2	0.02
Swallowing, independent	91.3	89.2	0.01
Eating, independent	99.9	99.4	<0.01
Urination, independent	99.5	97.6	<0.01
Defecation, independent	99.8	98.6	<0.01
Oral hygiene, independent	99.9	97.7	<0.01
Washing face, independent	99.7	92.8	<0.01
Hairdressing, independent	99.8	98.4	<0.01
Dressing upper body, independent	99.1	95.9	<0.01
Dressing lower body, independent	99.7	96.6	<0.01
Going outdoors, more than once a week	91.0	49.2	<0.01
(3) Cognitive functions			
Expression of one’s intention, possible	99.5	98.3	<0.01
Understanding daily routine, possible	99.9	99.7	0.09
Recalling one’s birthday or age, possible	99.8	99.6	0.39
Short-term memory (Recalling the events before the interview), possible	95.7	90.5	<0.01
Recalling one’s name, possible	100.0	100.0	–
Recalling the current season, possible	99.4	98.1	<0.01
Recalling where one is, possible	100.0	99.9	0.58
Wandering behavior, none (absence)	100.0	99.9	0.58
Difficulty to return home, none (absence)	99.8	99.5	0.07
(4) Psychiatric and behavioral symptoms			
Paranoia (delusion of theft and so on), none (absence)	98.8	98.7	0.60
Confabulation, none (absence)	98.5	98.1	0.26
Rapid changes in moods, none (absence)	97.8	97.2	0.17
Inverted circadian rhythm, none (absence)	99.5	99.5	0.73
Repeating the same story, none (absence)	95.7	94.6	0.09
Shouting out, none (absence)	98.9	98.8	0.70
Resisting to care, none (absence)	100.0	99.9	0.57
Restless, none (absence)	100.0	99.9	0.35
Elopement behavior, none (absence)	100.0	100.0	–
Collection mania, none (absence)	100.0	99.9	0.58
Violence, none (absence)	100.0	99.9	0.33
Severe memory loss, none (absence)	65.5	66.9	0.29
Inappropriate laughing, none (absence)	99.6	99.3	0.31
Selfish behaviors, none (absence)	99.8	99.8	1.00
Difficulty of communication (fluently and logically), None (absence)	99.6	99.6	0.88

ADL, activities of daily living.

**Table 2:** Association between shopping assistance and functional decline in all participants.

	OR	95% CI
Age (years)		
65–74	1.00 (ref)	
>85	1.40	1.22–1.61
Sex	0.51	0.44–0.59
(1) Physical functions		
Standing with feet without holding	1.40	1.13–1.74
Walking without holding	1.48	1.24–1.76
Standing on one leg without holding	1.38	1.18–1.61
Washing body	1.61	1.31–1.97
Cutting nails	1.82	1.52–2.19
Acuity	1.63	1.16–2.27
Hearing	1.20	1.04–1.39
(2) ADL functions		
Eating	7.15	0.92–55.82
Urination	2.91	1.36–6.19
Oral hygiene	14.08	1.91–103.96
Washing face	4.23	1.70–10.52
Dressing lower body	2.96	1.14–7.66
Going outdoors	7.26	6.02–8.76
(3) Cognitive functions		
Expression of one’s intention	3.15	1.43–6.93
Short-term memory (Recalling the events before the interview)	2.79	2.09–3.74
Recalling current season	2.33	1.13–4.80
(4) Psychiatric and behavioral symptoms		
Confabulation	1.66	1.00–2.75
Repeating the same story	1.55	1.14–2.11
Severe memory loss	1.19	1.03–1.37

**Table 3:** Association between shopping assistance and functional decline in male participants.

	OR	95% CI
Age (years)		
65–74	1.00 (ref)	
75–84	1.50	1.07–2.10
>85	1.80	1.29–2.49
(1) Physical functions		
Walking without holding	1.72	1.30–2.27
Cutting nails	1.44	1.04–1.99
Acuity	1.95	1.10–3.43
(2) ADL functions		
Oral hygiene	10.76	1.45–80.0
Washing face	4.36	1.02–18.70
Dressing lower body	9.74	1.31–72.51
Going outdoors	8.82	6.19–12.58
(3) Cognitive functions		
Short-term memory (Recalling the events before the interview)	2.53	1.49–4.29
(4) Psychiatric and behavioral symptoms		
Severe memory loss	1.32	1.02–1.71

OR, odds ratio; CI, confidence interval; ADL, activities of daily living; ref, reference category.

**Table 4:** Association between shopping assistance and functional decline in female participants.

	OR	95% CI
Age (years)		
65–74	1.00 (ref)	
>85	1.49	1.28–1.75
(1) Physical functions		
Standing with feet without holding	1.43	1.12–1.83
Walking without holding	1.42	1.16–1.74
Standing on one leg without holding	1.55	1.28–1.89
Washing body	1.86	1.46–2.37
Cutting nails	2.22	1.79–2.75
(2) ADL functions		
Washing face	4.87	1.51–15.68
Going outdoors	6.95	5.58–8.66
(3) Cognitive functions		
Expression of one’s intention	6.73	1.94–23.39
Short-term memory (Recalling the events before the interview)	3.38	2.42–4.73
(4) Psychiatric and behavioral symptoms		
Repeating the same story	1.82	1.30–2.54

OR, odds ratio; CI, confidence interval; ADL, activities of daily living; ref, reference category.

## Data Availability

The data that support the findings of this study are available from the local government of Koriyama City, Fukushima, but restrictions apply to the availability of these data, which were used under license for the current study and so are not publicly available. The data are, however, available from the authors upon reasonable request and with the permission of the local government and Fukushima Medical University Ethics Committee.

## Supplementary Materials

The following supporting information can be downloaded at https://www.mdpi.com/article/10.3390/geriatrics9060162/s1: Table S1: Significant factors associated with shopping assistance for participants requiring supervision or partial dependence (ref: Independent); Table S2: Significant factors associated with shopping assistance for totally dependent participants (ref: Independent); Table S3: Significant factors associated with shopping assistance for male participants requiring supervision or partial dependence (ref: Independent); Table S4: Significant factors associated with shopping assistance for totally dependent male participants (ref: Independent); Table S5: Significant factors associated with shopping assistance for female participants requiring supervision or partial dependence (ref: Independent); Table S6: Significant factors associated with shopping assistance for totally dependent female participants (ref: Independent).
