# Supplementary material for: Association Between Shopping Assistance and Functional Decline in Older Residents with Support Levels Under the Long-Term Care Insurance System in Japan: A Retrospective, Cross-Sectional Study
Source: Geriatrics (Basel). 2024 Dec 14;9(6):162. doi: 10.3390/geriatrics9060162 (PMC11728436; doi:10.3390/geriatrics9060162)
Supplement: Supplementary file 1 [file geriatrics-09-00162-s001.zip › geriatrics-3237986-supplementary.pdf]

Table S1. Significant factors associated with shopping assistance for participants requiring supervision or partial dependence (ref: Independent).

	OR	95% CI (LL-HL)		p-value
Sex,				
Male (ref)	1.00			
Female	1.33	1.07	1.67	**0.012**
Health condition and functional activities				
(1) Physical functions				
Walking,				
Possible without holding (ref)	1.00			
Need assistance	1.53	1.20	1.94	**0.001**
Impossible	2.11	0.57	7.77	0.262
Maintaining a standing on one leg,				
Possible without holding (ref)	1.00			
Need assistance	1.29	1.02	1.68	**0.034**
Impossible	2.59	0.78	8.65	0.122
Cutting nails,				
Independent (ref)	1.00			
Partially dependent	1.28	0.98	1.66	0.069
Totally dependent	2.93	1.68	5.08	**<0.001**
Acuity,				
Normal (possible over 1 m distance) (ref)	1.00			
Possible at 1 m distance	1.25	0.66	2.39	0.493
Possible at close to one's eye	1.06	0.54	2.07	0.869
Almost blind	8.69	1.84	41.17	**0.006**
Impossible to evaluate	NA	NA	NA	NA
(2) ADL functions				
Oral hygiene,				
Independent (ref)	1.00			
Partially dependent	27.19	3.28	225.20	**0.002**
Totally dependent	NA	NA	NA	NA
Going outdoors,				
More than once a week (ref)	1.00			
More than once a month	3.88	3.05	4.93	**<0.001**
Less than once a month	8.87	3.54	22.22	**<0.001**
(3) Cognitive functions				
Short-term memory,				
Possible (ref)	1.00			
Impossible	1.68	1.11	2.55	**0.014**
(4) Psychiatric and behavioral symptoms				
Rapid changes in moods,				
None (absence) (ref)	1.00			
Occasionally presence	2.55	1.01	6.45	**0.048**
Often presence	1.47	0.62	3.49	0.383
Repeating the same story,				
None (absence) (ref)	1.00			
Occasionally presence	0.68	0.27	1.69	0.407
Often presence	1.76	1.07	2.87	**0.025**

Bold indicates significant variables.

Table S2. Significant factors associated with shopping assistance for totally dependent participants (ref: Independent).

	OR	95% CI (LL-HL)		p-value
Age				
65–74 (ref)	1.00			
75–84	1.28	1.01	1.63	**0.040**
>85	1.97	1.55	2.51	**<0.001**
Sex,				
Male (ref)	1.00			
Female	0.38	0.33	0.45	**<0.001**
Health condition and functional activities				
(1) Physical functions				
Contracture,				
None (absence) (ref)	1.00			
Exist (presence)	0.77	0.61	0.98	**0.033**
Maintaining a standing with feet,				
Possible without holding (ref)	1.00			
Need assistance	1.44	1.14	1.82	**0.002**
Impossible	NA	NA	NA	0.977
Walking,				
Possible without holding (ref)	1.00			
Need assistance	1.48	1.23	1.79	**<0.001**
Impossible	2.16	0.69	6.70	0.185
Maintaining a standing on one leg,				
Possible without holding (ref)	1.00			
Need assistance	1.40	1.17	1.67	**<0.001**
Impossible	2.14	0.71	6.42	0.176
Washing body,				
Independent (ref)	1.00			
Partially dependent	1.89	1.47	2.43	**<0.001**
Totally dependent	2.92	0.34	24.94	0.328
Not conduction	0.79	0.52	1.19	0.260
Cutting nails,				
Independent (ref)	1.00			
Partially dependent	1.60	1.29	1.97	**<0.001**
Totally dependent	4.32	2.68	6.95	**<0.001**
Acuity,				
Normal (possible over 1 m distance) (ref)	1.00			
Possible at 1 m distance	1.66	1.01	2.73	**0.046**
Possible at close to one's eye	1.50	0.90	2.48	0.119
Almost blind	2.38	0.49	11.60	0.285
Impossible to evaluate	-	-	-	
Hearing,				
Normal (ref)	1.00			
Occasionally difficult	1.20	1.03	1.41	**0.023**
Possible by loud voice	1.80	1.07	3.04	**0.027**
Almost deaf	NA	NA	NA	0.972
Impossible to evaluate	NA	NA	NA	NA
(2) ADL functions				
Urination,				

	OR	95% CI (LL-HL)		p-value
Independent (ref)	1.00			
Supervision required	1.53	0.25	9.49	0.650
Partially dependent	3.28	1.37	7.86	**0.008**
Totally dependent	NA	NA	NA	0.985
Oral hygiene,				
Independent (ref)	1.00			
Partially dependent	15.24	2.00	116.21	**0.009**
Totally dependent	NA	NA	NA	NA
Washing face,				
Independent (ref)	1.00			
Partially dependent	3.06	1.15	8.20	**0.026**
Totally dependent	1.29	NA	NA	1.000
Going outdoors,				
More than once a week (ref)	1.00			
More than once a month	6.80	5.58	8.29	**<0.001**
Less than once a month	58.10	25.37	133.06	**<0.001**
(3) Cognitive functions				
Expression of one's intention,				
Possible (ref)	1.00			
Occasionally impossible	3.48	1.53	7.92	**0.003**
Almost impossible	NA	NA	NA	NA
Impossible	NA	NA	NA	NA
Short-term memory,				
Possible (ref)	1.00			
Impossible	2.80	2.05	3.83	**<0.001**
Recalling the current season,				
Possible (ref)	1.00			
Impossible	2.61	1.21	5.60	**0.014**
(4) Psychiatric and behavioral symptoms				
Repeating the same story,				
None (absence) (ref)	1.00			
Occasionally presence	0.66	0.34	1.30	0.231
Often presence	2.03	1.38	2.99	**<0.001**
Shouting out,				
None (absence) (ref)	1.00			
Occasionally presence	2.24	0.83	6.04	0.112
Often presence	0.33	0.11	0.99	**0.047**
Severe memory loss,				
None (absence) (ref)	1.00			
Occasionally presence	1.03	0.84	1.26	0.813
Often presence	1.45	1.20	1.76	**<0.001**
Inappropriate laughing,				
None (absence) (ref)	1.00			
Occasionally presence	0.16	0.01	4.17	0.267
Often presence	3.09	1.03	9.22	**0.043**

Bold indicates significant variables.

Table S3. Significant factors associated with shopping assistance for male participants requiring supervision or partial dependence (ref: Independent).

	OR	95% CI (LL-HL)		p-value
Health condition and functional activities				
(1) Physical functions				
Maintaining a sitting Position,				
Possible without holding (ref)	1.00			
Using upper limb (grabbing a bar)	1.33	0.79	2.25	0.276
Need assistance	2.92	1.19	7.18	**0.020**
Impossible	0.83	NA	NA	1.000
Walking,				
Possible without holding (ref)	1.00			
Need assistance	2.12	1.22	3.69	**0.008**
Impossible	9.04	0.78	104.30	0.078
Acuity,				
Normal (possible over 1 m distance) (ref)	1.00			
Possible at 1 m distance	0.60	0.16	2.35	0.465
Possible at close to one's eye	4.22	1.07	16.66	**0.040**
Almost blind	NA	NA	NA	0.981
Impossible to evaluate	-	-	-	
(2) ADL functions				
Oral hygiene,				
Independent (ref)	1.00			
Partially dependent	32.70	3.48	307.39	**0.002**
Totally dependent	NA	NA	NA	NA
Going outdoors,				
More than once a week (ref)	1.00			
More than once a month	4.22	2.50	7.11	**<0.001**
Less than once a month	2.32	0.35	15.36	0.385
(3) Cognitive functions				
Repeating the same story,				
None (absence) (ref)	1.00			
Occasionally presence	NA	NA	NA	0.986
Often presence	3.70	1.22	11.22	**0.021**

Bold indicates significant variables.

Table S4. Significant factors associated with shopping assistance for totally dependent male participants (ref: Independent).

	OR	95% CI (LL-HL)		p-value
Age				
65–74 (ref)	1.00			
75–84	1.71	1.18	2.50	**0.005**
>85	2.01	1.37	2.94	**<0.001**
Health condition and functional activities				
(1) Physical functions				
Walking,				
Possible without holding (ref)	1.00			
Need assistance	1.49	1.04	2.13	**0.030**
Impossible	1.60	0.17	15.17	0.680
Washing body,				
Independent (ref)	1.00			
Partially dependent	1.54	0.98	2.42	0.061
Totally dependent	NA	NA	NA	0.985
Not conduction	0.41	0.21	0.81	**0.010**
Cutting nails,				
Independent (ref)	1.00			
Partially dependent	1.04	0.72	1.52	0.821
Totally dependent	3.19	1.27	8.05	**0.014**
Acuity,				
Normal (possible over 1 m distance) (ref)	1.00			
Possible at 1 m distance	0.80	0.37	1.73	0.574
Possible at close to one's eye	3.68	1.25	10.83	**0.018**
Almost blind	NA	NA	NA	0.982
Impossible to evaluate	-	-	-	
(2) ADL functions				
Oral hygiene,				
Independent (ref)	1.00			
Partially dependent	12.02	1.44	100.07	**0.022**
Totally dependent	NA	NA	NA	NA
Going outdoors,				
More than once a week (ref)	1.00			
More than once a month	7.72	5.26	11.33	**<0.001**
Less than once a month	36.84	11.10	122.26	**<0.001**
(3) Cognitive functions				
Short-term memory,				
Possible (ref)	1.00			
Impossible	2.63	1.48	4.68	**0.001**
(4) Psychiatric and behavioral symptoms				
Severe memory loss,				
None (absence) (ref)	1.00			
Occasionally presence	1.09	0.75	1.57	0.662
Often presence	1.55	1.09	2.19	**0.014**

Bold indicates significant variables.

Table S5. Significant factors associated with shopping assistance for female participants requiring supervision or partial dependence (ref: Independent).

	OR	95% CI (LL-HL)		p-value
Health condition and functional activities				
(1) Physical functions				
Walking,				
Possible without holding (ref)	1.00			
Need assistance	1.45	1.11	1.89	**0.006**
Impossible	1.84	0.33	10.46	0.490
Maintaining a standing on one leg,				
Possible without holding (ref)	1.00			
Need assistance	1.48	1.12	1.95	**0.006**
Impossible	2.56	0.73	8.92	0.141
Cutting nails,				
Independent (ref)	1.00			
Partially dependent	1.52	1.13	2.06	**0.006**
Totally dependent	3.72	1.99	6.96	**<0.001**
Acuity,				
Normal (possible over 1 m distance) (ref)	1.00			
Possible at 1 m distance	1.86	0.83	4.16	0.133
Possible at close to one's eye	0.64	0.29	1.42	0.272
Almost blind	5.68	1.13	28.65	**0.035**
Impossible to evaluate	-	-	-	
(2) ADL functions				
Going outdoors,				
More than once a week (ref)	1.00			
More than once a month	3.82	2.90	5.03	**<0.001**
Less than once a month	13.04	3.82	44.54	**<0.001**
(3) Cognitive functions				
Expression of one's intention,				
Possible (ref)	1.00			
Occasionally impossible	5.05	1.15	22.28	**0.033**
Almost impossible	NA	NA	NA	NA
Impossible	NA	NA	NA	NA
Short-term memory,				
Possible (ref)	1.00			
Impossible	1.94	1.20	3.11	**0.007**

Bold indicates significant variables.

Table S6. Significant factors associated with shopping assistance for totally dependent female participants (ref: Independent).

	OR	95% CI (LL-HL)		p-value
Age				
65–74 (ref)	1.00			
75–84	1.10	0.80	1.51	0.564
>85	1.89	1.37	2.61	**<0.001**
Health condition and functional activities				
(1) Physical functions				
Maintaining a standing with feet,				
Possible without holding (ref)	1.00			
Need assistance	1.52	1.16	1.99	**0.002**
Impossible	NA	NA	NA	0.968
Walking,				
Possible without holding (ref)	1.00			
Need assistance	1.47	1.18	1.85	**0.001**
Impossible	3.44	0.76	15.50	0.108
Maintaining a standing on one leg,				
Possible without holding (ref)	1.00			
Need assistance	1.51	1.21	1.90	**<0.001**
Impossible	1.49	0.46	4.81	0.510
Washing body,				
Independent (ref)	1.00			
Partially dependent	1.99	1.47	2.71	**<0.001**
Totally dependent	2.00	0.23	17.64	0.533
Not conduction	1.08	0.64	1.80	0.784
Cutting nails,				
Independent (ref)	1.00			
Partially dependent	1.89	1.46	2.43	**<0.001**
Totally dependent	5.02	2.86	8.82	**<0.001**
Acuity,				
Normal (possible over 1 m distance) (ref)	1.00			
Possible at 1 m distance	2.67	1.35	5.32	**0.005**
Possible at close to one's eye	0.97	0.52	1.80	0.919
Almost blind	1.40	0.24	8.20	0.708
Impossible to evaluate	NA	NA	NA	NA
(2) ADL functions				
Washing face,				
Independent (ref)	1.00			
Partially dependent	3.58	1.01	12.74	**0.049**
Totally dependent	1.93	NA	NA	0.999
Going outdoors,				
More than once a week (ref)	1.00			
More than once a month	6.63	5.23	8.39	**<0.001**
Less than once a month	76.56	24.02	244.02	**<0.001**
(3) Cognitive functions				
Expression of one's intention,				
Possible (ref)	1.00			
Occasionally impossible	6.16	1.61	23.53	**0.008**
Almost impossible	NA	NA	NA	NA

	OR	95% CI (LL-HL)		p-value
Impossible	NA	NA	NA	NA
Short-term memory,				
Possible (ref)	1.00			
Impossible	3.03	2.06	4.44	**<0.001**
Recalling the current season,				
Possible (ref)	1.00			
Impossible	2.97	1.16	7.58	**0.023**
(4) Psychiatric and behavioral symptoms				
Inverted circadian rhythm,				
None (absence) (ref)	1.00			
Occasionally presence	0.74	0.13	4.18	0.734
Often presence	0.18	0.04	0.87	**0.033**
Repeating the same story,				
None (absence) (ref)	1.00			
Occasionally presence	0.66	0.31	1.43	0.293
Often presence	2.44	1.57	3.80	**<0.001**
Shouting out,				
None (absence) (ref)	1.00			
Occasionally presence	1.93	0.45	8.24	0.376
Often presence	0.14	0.02	0.83	**0.031**
Severe memory loss,				
None (absence) (ref)	1.00			
Occasionally presence	0.98	0.76	1.25	0.845
Often presence	1.40	1.11	1.77	**0.005**
Inappropriate laughing,				
None (absence) (ref)	1.00			
Occasionally presence	NA	NA	NA	0.976
Often presence	3.42	1.04	11.28	**0.043**

Bold indicates significant variables.
